# A Configurable Real2Sim Bimanual Teleoperation Framework for Surgical Robotic Tool Design and Evaluation

**DOI:** 10.1002/rcs.70235

**Published:** 2026-10-09

**Authors:** Teng Li, Sunny Zhang, Cate Balasubramanian, Thomas Looi, Dale J. Podolsky

**Affiliations:** ^1^ The Wilfred and Joyce Posluns Center for Image Guided Innovation and Therapeutic Intervention (PCIGITI) The Hospital for Sick Children (SickKids) Toronto Ontario Canada; ^2^ The Division of Plastic, Reconstructive & Aesthetic Surgery, Department of Surgery University of Toronto Toronto Ontario Canada; ^3^ The Department of Mechanical and Industrial Engineering (MIE), Faculty of Applied Science and Engineering University of Toronto Toronto Ontario Canada; ^4^ The Division of Engineering Science, Faculty of Applied Science and Engineering University of Toronto Toronto Ontario Canada; ^5^ The Department of Systems Design Engineering Faculty of Engineering, University of Waterloo Waterloo Ontario Canada; ^6^ The Department of Otolaryngology ‐ Head & Neck Surgery Temerty Faculty of Medicine, University of Toronto Toronto Ontario Canada

**Keywords:** bimanual teleoperation, cleft surgery, real2sim, robotic tool design, surgical robot

## Abstract

**Background:**

During surgical robotic tool development, teleoperation can provide a valuable means of evaluating tool performance and informing iterative design improvements.

**Methods:**

We propose a compact framework for establishing real2sim bimanual teleoperation systems. Using this framework, an Omni‐to‐Unity system was established to enable bimanual teleoperation of simulated robotic tools using two portable haptic devices. A dVRK‐to‐Unity system was also implemented to evaluate the framework with a different leader robot. An application scenario was presented to illustrate its use in robotic tool design.

**Results:**

Both systems were integrated with simulated surgical tasks and operated without observed system failures during the reported experiments. The experiments showed the applicability of the framework to different leader robots and simulated robotic tools with different kinematic configurations.

**Conclusions:**

The proposed framework provides a compact approach to establishing bimanual real2sim teleoperation systems and facilitates the evaluation of different robotic tool designs through simulated surgical tasks.

## 1 Introduction

Robot‐assisted surgery has increasingly been adopted for minimally invasive surgical procedures as robotic technologies have continued to advance [1, 2]. Reported clinical benefits of robot‐assisted surgery include shorter recovery times and hospital stays, as well as reduced postoperative pain in selected procedures [3]. In 2000, the da Vinci Surgical System (dVSS) received approval from the U.S. Food and Drug Administration (FDA) for clinical use. Since then, robot‐assisted minimally invasive surgery (MIS) has been applied across a range of surgical specialties, providing surgeons with enhanced dexterity for instrument manipulation within confined anatomical spaces [4]. The increasing application of robot‐assisted MIS has also motivated the development of robotic tools tailored to different surgical procedures and anatomical constraints [5, 6, 7]. Applications of the dVSS and related robotic technologies have expanded from laparoscopic procedures to various areas such as prostate surgery, mitral valve repair, gynaecological surgery, and orthopaedic surgery [8, 9].

Cleft palate repair is a transoral surgical procedure that has been investigated as a potential application for robot assistance. The small infant oral cavity presents particular challenges for robotic intervention because surgical instruments must be manipulated dexterously within a confined workspace while interacting with delicate tissue [10, 11]. The da Vinci Research Kit (dVRK), a research version of the dVSS, has previously been evaluated for cleft palate repair using a high‐fidelity surgical simulator [11]. However, the standard 8 mm EndoWrist instruments are relatively large for the infant oral workspace, and the multiport architecture of the dVRK was originally designed for procedures involving larger body cavities rather than transoral access, where instrument triangulation is constrained [11]. These limitations motivate the development of transoral robotic systems (TORS) and miniaturised robotic tools specifically designed for procedures within the oral cavity. Our previous studies developed 3‐mm‐diameter robotic tools based on the standard 8 mm dVRK instruments and evaluated their performance in simulated cleft palate surgery [6, 7, 12]. These studies provide motivation for continued development and evaluation of robotic tool designs and system architectures tailored to cleft palate repair and other transoral procedures.

During the development of a new robotic tool, virtual evaluation can provide a practical means of examining candidate designs before physical prototypes are manufactured. In particular, teleoperating a simulated robotic tool during virtual surgical tasks allows designers to observe its kinematic behaviour and interaction with the simulated surgical environment, thereby providing complementary information to conventional analyses such as workspace and dexterity evaluation. One approach is to use the dVRK master tool manipulators (MTMs) as leader devices for teleoperating simulated robotic tools. For example, Fontanelli et al. [13] developed a V‐REP simulator in which two virtual patient side manipulators (PSMs) were teleoperated using two physical MTMs. However, access to a dVRK may be limited, and a framework that is not restricted to a particular leader platform could provide greater flexibility for robotic tool evaluation. Such a framework would allow different available leader devices to teleoperate simulated robotic tool designs during virtual evaluation tasks.

In telerobotics, real‐to‐real (real2real) teleoperation, in which a physical leader device controls a physical follower robot, has been extensively investigated [14, 15, 16]. Such systems often incorporate control strategies to address robot dynamics, communication, tracking, and physical interaction [17, 18, 19, 20, 21, 22]. For example, Naceri et al. [23] developed a virtual reality (VR)‐based interface, *Vicarios*, in which a user wearing a head‐mounted display teleoperates a physical 6‐degree‐of‐freedom (DOF) robotic arm using an ungrounded HTC Vive Pro motion controller. The pose of the motion controller is mapped to the 6D pose of the follower robot using a velocity‐based control strategy with proportional control. A digital representation of the remote physical robot is simultaneously rendered within the VR environment.

Real2sim and surgical teleoperation systems with different leader‐follower configurations have also been reported. Ye et al. [24] developed a system in which a 6‐DOF Phantom device teleoperated a virtual 6‐DOF robot designed for femur bone fracture reduction surgery. The virtual robot consisted of six prismatic joints, and an analytical inverse kinematics (IK) solution derived for its specific kinematic structure was used to map the Cartesian pose of the Phantom device to the virtual robot [25]. Dhivin et al. [26] developed a bilateral tele‐haptic interface in which a 6‐DOF haptic device controlled the 3D Cartesian position of a 5‐DOF robotic manipulator with an analytically derived IK solution. Wang et al. [27] and Nasiri et al. [28] developed teleoperation approaches for robot‐assisted MIS incorporating algorithm‐based remote centre of motion (RCM) constraints. Garcia‐Cardenas et al. [29] developed a teleoperation system for soft‐tissue incision in which a haptic device controlled the 3D position of a physical 6‐DOF robot while a joystick controlled its orientation. Collectively, these studies illustrate different teleoperation strategies developed for specific combinations of leader devices, follower robots, and surgical applications.

Although teleoperation has been extensively investigated, much of the existing literature focuses on real2real scenarios, for which both the kinematics and physical behaviour of the follower robot must be considered. Consequently, many existing approaches incorporate control architectures tailored to the characteristics and requirements of a particular robotic system or task [17, 18, 19, 20, 21, 22]. The requirements are different during the early‐stage evaluation of a robotic tool design, where a physical follower may not yet be available and the tool may initially be represented primarily by its kinematic model. Furthermore, kinematic parameters (e.g., link dimensions, joint types, and the number of joints) may change across design iterations. This motivates a compact real2sim teleoperation framework in which the follower kinematics can be readily reconfigured, allowing different simulated robotic tool designs to be evaluated without developing a dedicated teleoperation implementation for each design.

In our previous work [30], we presented a framework for establishing a real2sim teleoperation system comprising one haptic device and one simulated robotic tool. In the present work, the framework is extended to bimanual teleoperation involving two leader devices and two simulated robotic tools. The framework uses configurable Denavit‐Hartenberg (DH) parameters and adjustable initial joint configurations to define the kinematics of the simulated robotic tools, allowing these parameters to be modified when evaluating different tool designs. Based on the proposed framework, two bimanual teleoperation systems were implemented: (1) a portable Omni‐to‐Unity system using two haptic devices and (2) a dVRK‐to‐Unity system using two dVRK MTMs. A series of experiments and application demonstrations were conducted to evaluate the implemented systems and examine the framework under different leader and follower configurations. The main contributions of this work are summarised as follows:

- A compact framework for real2sim bimanual teleoperation is proposed, in which the kinematics of serial simulated robotic tools are defined using adjustable DH parameters.
- The framework accommodates different leader devices and simulated robotic tool configurations through configurable coordinate transformations, motion scaling, initial joint configurations, and follower kinematic parameters.
- Two bimanual teleoperation systems are implemented and evaluated, together with application demonstrations involving robotic tool design, different follower kinematics, and haptic feedback.

## 2 Materials and Methods

This study did not involve human participants or animals. Therefore, ethical approval and informed consent were not required.

### 2.1 Kinematic Modelling

The base frames of both the real robots and the simulated robotic tools need to be defined to determine the transformation matrix from the real robot's base frame to the corresponding simulated robotic tool's base frame. For each simulated robotic tool, the complete forward kinematics (FK) must also be derived. First, coordinate frames are assigned to each joint of the simulated robotic tool. The corresponding Denavit‐Hartenberg (DH) parameters are then determined for deriving the FK and Jacobian matrix. As a specific example, the coordinate frames assigned to the two simulated robotic tools used in this work are shown in Figure 1. These robotic tools represent our initial designs for cleft palate surgery and are evaluated within a virtual environment for design assessment and performance characterisation.

**FIGURE 1:**
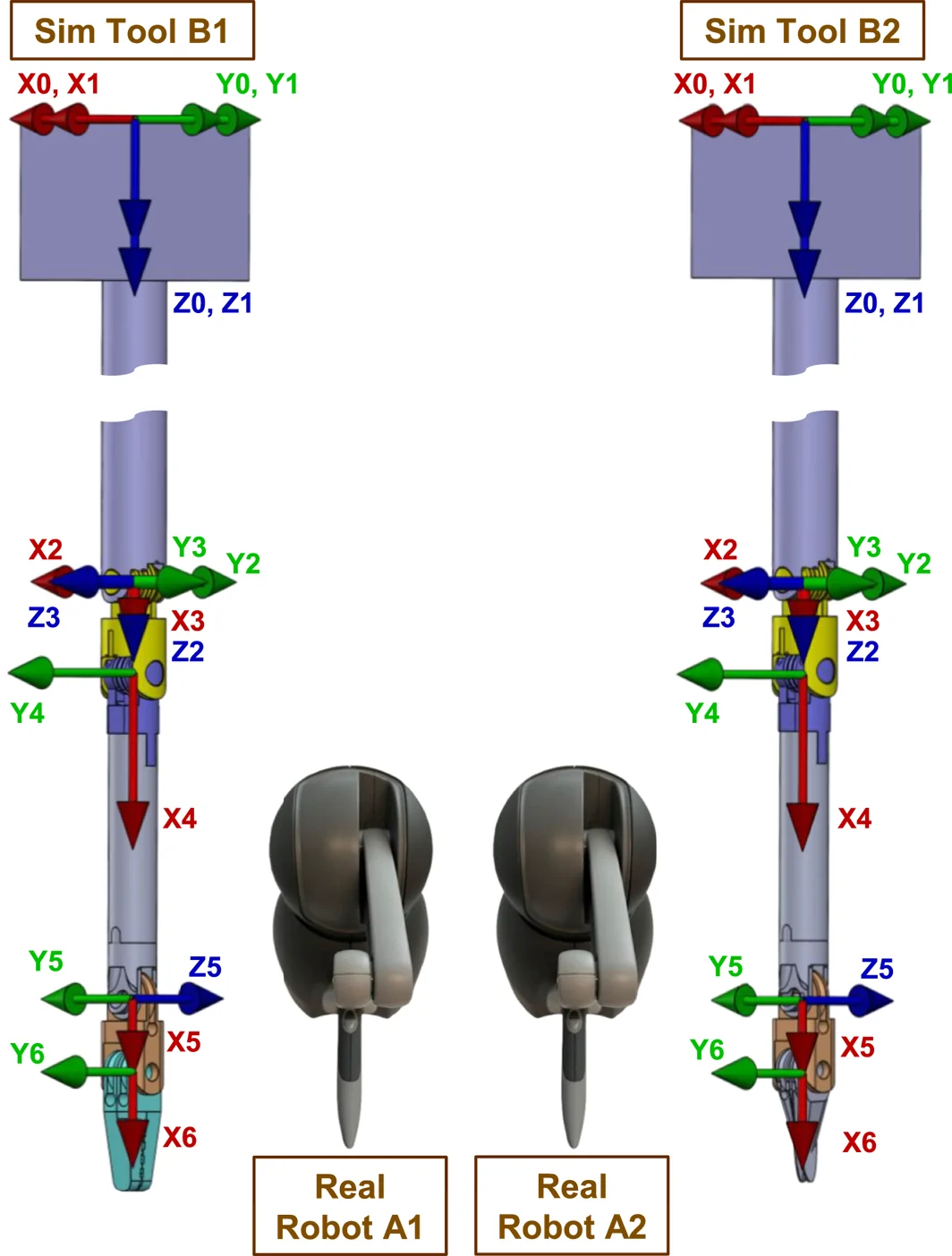
Two 6‐DOF simulated robotic tools (PRRRRR) with coordinate frames assigned to their respective joints. Sim Tool B1 is a grasper, whereas Sim Tool B2 is a pair of scissors; they are teleoperated by Real Robots A1 and A2, respectively.

Based on the assigned coordinate frames, the corresponding DH parameters are determined and provided in Table 1. The two simulated robotic tools illustrated in Figure 1 share the same frame assignments and DH parameters because they have the same overall kinematic structure, while the primary difference is that Sim Tool B1 has grasper jaws, whereas Sim Tool B2 has scissor jaws.

**TABLE 1:** Denavit‐Hartenberg (DH) parameters for the simulated 6‐DOF robotic tools.

Frame	Type	i	$a_{i-1}\left(m\right)$	$\alpha_{i-1}\left(rad\right)$	$d_{i}\left(m\right)$	$\theta_{i}\left(rad\right)$
Joint 1	2	1	0	0	$d_{1}$	0
Joint 2	1	2	0	0	$L_{2}$	$q_{2}+\pi /2$
Joint 3	1	3	0	π/2	0	$q_{3}$
Joint 4	1	4	$L_{3}$	π/2	0	$q_{4}$
Joint 5	1	5	$L_{4}$	π/2	0	$q_{5}$
Joint 6 (EE)	1	6	$L_{5}$	−π/2	0	$q_{6}$

*Note:* Type 1, revolute joint; Type 2, prismatic joint; $d_{1}$, displacement variable of the prismatic joint; $L_{2}∼L_{5}$, link lengths.

Based on the DH parameters provided in Table 1, the homogeneous transformation matrix between two adjacent frames, following the modified DH convention, is given by

(1) Tii−1=cθi−sθi0ai−1sθicαi−1cθicαi−1−sαi−1−sαi−1disθisαi−1cθisαi−1cαi−1cαi−1di0001

where c and s denote cos(⋅) and sin(⋅), respectively, and i=1,2,…,n denotes the i‐th link/frame of the robot.

The homogeneous transformation from the base frame {0} to the end‐effector (EE) frame {6} can then be obtained using Equation (2) for Sim Tools B1 and B2 based on their respective DH parameters.

(2) TB.eeB=Tee0=T10T21T32T43T54T65

where TB.eeB represents the EE pose of Sim Tool B expressed with respect to its base frame {0}. Equation (2) therefore represents the FK of Sim Tool B. For compactness, B is used hereafter to collectively denote B1 and B2, and A is used to collectively denote A1 and A2, unless otherwise specified.

For the real robot, we assume that the EE pose of Real Robot A can be accessed in real time and is denoted by TA.eeA, or $T_{A.ee}$ for compactness.

Based on the base‐frame definitions of the real robot and simulated robotic tool, a constant transformation matrix from the base frame of Real Robot A to that of Sim Tool B is defined as TAB. The EE pose of Real Robot A can then be expressed in the coordinate system of Sim Tool B as

(3) TA.eeB=TABTA.ee

where $T_{A.ee}$ represents the EE pose of Real Robot A expressed in its own base frame in SE(3) format, and TA.eeB represents the same EE pose expressed in the base frame of Sim Tool B.

### 2.2 Full 6D Pose Mapping

The 3D position and 3D orientation mappings, which together define the full 6D pose mapping between the real robot and simulated robotic tool, are performed separately. A relative mapping strategy is used for position. Specifically, the displacement of the real robot's EE relative to a reference position is first calculated, scaled, and then applied to the EE position of the simulated robotic tool. The desired position is given by

(4) tB.des=(tA.eeB−tA.frozB)∗s+tB.froz

where s is the motion scaling factor, tA.frozB is the position of Real Robot A recorded in the coordinate system of Sim Tool B at the instant when the button or foot pedal is pressed, and $t_{B.froz}$ is the corresponding EE position of Sim Tool B recorded at the same instant. These reference positions allow the relative translational motion of the real robot to be scaled and mapped to the simulated robotic tool. The resulting desired 3D position of Sim Tool B is denoted by $t_{B.des}$.

Similar to the position mapping, the 3D orientation is mapped relatively. First, the relative orientation of Real Robot A with respect to its initial orientation is calculated as

(5) ΔBRA.ee=RA.eeBRA.init−1B

where RA.initB represents the initial orientation of Real Robot A expressed in the coordinate system of Sim Tool B.

The relative orientation ΔRA.eeB of Real Robot A is then applied to the initial orientation of Sim Tool B to obtain its desired orientation,

(6) RB.des=ΔRA.eeBRB.init

where $R_{B.init}$ and $R_{B.des}$ denote the initial and desired orientations of Sim Tool B, respectively.

### 2.3 Inverse Kinematics

By combining the mapped position and orientation in SE(3) form, the desired Cartesian EE pose of the simulated robotic tool is obtained and subsequently used to determine the corresponding joint configuration through inverse kinematics (IK). IK solutions can generally be obtained using either analytical or numerical approaches. When available, an analytical IK solution can provide computational advantages. However, it is typically derived for a specific robot kinematic structure. In this work, the framework is intended to accommodate serial robotic tools with different kinematic configurations and therefore requires an IK solver that can be applied without deriving a new analytical solution for each tool design. Moreover, the complexity of deriving analytical IK solutions generally increases with the number of robot DOFs and the complexity of the kinematic structure [31].

Because the kinematic configuration of a robotic tool may change across design iterations, repeatedly deriving an analytical IK solution for each design would limit the flexibility of the framework. Therefore, a numerical IK approach is adopted and implemented for the simulated robotic tools [30, 31]. Specifically, the implemented numerical IK solver uses a Gauss–Newton iteration with Levenberg–Marquardt damping, expressed as

(7) $${\Delta q}_{k}={\left(J^{T}WJ+\lambda I_{6\times 6}\right)}^{-1}{J^{T}We}_{k}$$

where the subscript k denotes the k‐th iteration of the IK algorithm, with $k\in \left[0,k_{\max}\right]$ and $k_{\max}=500$ in this work. ${\Delta q}_{k}$ represents the joint‐space update calculated from the current joint configuration. W is a diagonal weighting matrix, set to the identity matrix in this work because both EE position and orientation are considered. λ>0 is a variable damping factor (λ=0.1 in this work), and $e_{k}$ is the 6D pose error vector between the desired and current EE poses, with $\|e_{k}\|<10^{-10}$ used as the IK convergence threshold. The Jacobian J is expressed in the EE frame. The body Jacobian, expressed in the robot EE frame, can be obtained from the space Jacobian, expressed in the robot base frame, as

(8) Jee=ReeT000ReeT0J0

where Ree0 is the rotation matrix representing the orientation of the robot EE with respect to the robot base frame {0}. Further details of the numerical IK algorithm are provided in our previous work [30].

### 2.4 Jacobian Calculation

Jacobian calculation is an essential component of the numerical IK solver, as shown in Equation (7). A common approach to Jacobian calculation uses a link‐by‐link recursive formulation based on linear and angular velocities [32]. In this work, a differential transformation method [33] is employed to calculate the Jacobian directly from the homogeneous transformation matrices Tii−1 defined in Equation (1).

First, the homogeneous transformation matrices Tni(i=1,2,…,n−1) representing the EE frame with respect to each intermediate frame are obtained as

(9) T21T32⋯Tii−1Ti+1i⋯Tn−1n−2Tnn−1=Tn1T32⋯Tii−1Ti+1i⋯Tn−1n−2Tnn−1=Tn2⋯Tii−1Ti+1i⋯Tn−1n−2Tnn−1=Tni−1Ti+1i⋯Tn−1n−2Tnn−1=Tni⋯Tn−1n−2Tnn−1=Tnn−2Tnn−1=Tnn−1

Each transformation matrix Tni can be expressed in column vector form as

(10) Tni=noap0001

where n,o,a,p denote the four column vectors of the transformation matrix.

For the i‐th joint, if it is revolute, the corresponding column of the body Jacobian is given by

(11) Jiee=JlieeJaiee=(p×n)z(p×o)z(p×a)znzozaz

where n,o,a,p are the column vectors of the corresponding transformation matrix Tni obtained from Equation (9). Jliee and Jaiee denote the linear‐ and angular‐velocity components, respectively, of the i‐th column of the body Jacobian. Here, the EE is assumed to coincide with the n‐th frame, and the superscript ee is used instead of n for clarity.

For the i‐th joint, if it is prismatic, the corresponding column of the body Jacobian is given by

(12) Jiee=JlieeJaiee=nzozaz000

Using Equations ((9), (10), (11), (12)), all columns of the body Jacobian can be obtained. If required, the space Jacobian can subsequently be computed from the body Jacobian using the relationship given in Equation (8).

### 2.5 Framework of a Bimanual Teleoperation System

Algorithm 1 **Framework of Bimanual Teleoperation**

1

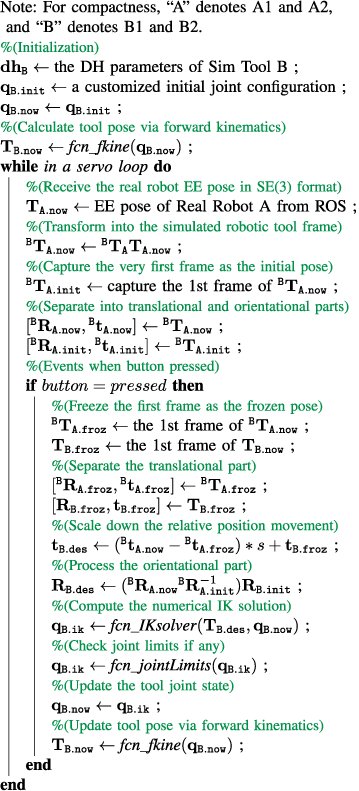

The proposed framework for establishing a real2sim bimanual teleoperation system is illustrated in Figure 2 and Algorithm 1. The two robot‐to‐tool pairs, A1‐B1 and A2‐B2, are established using the same procedure and operate in parallel. For each pair, the real robot publishes its real‐time Cartesian EE pose to a ROS topic, which is subscribed to by a real2sim kernel implemented in C++ for subsequent processing. Within the kernel, the EE pose of the real robot is first transformed into the coordinate system of the corresponding simulated robotic tool using Eqn. (3). The full 6D pose‐mapping method is then applied to determine the desired EE pose of the simulated robotic tool. Based on this desired pose, the numerical IK solver calculates the corresponding joint configuration [30], which is used to update the joint state of the simulated robotic tool.

**FIGURE 2:**
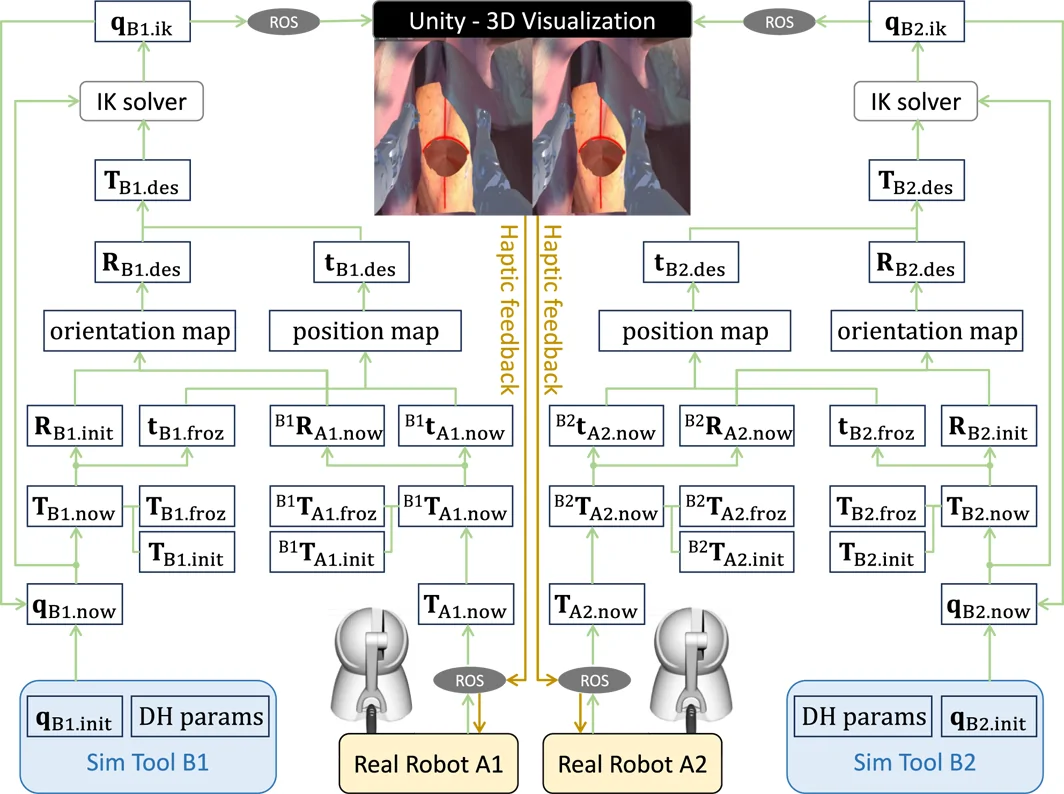
Framework of the real2sim bimanual teleoperation system. The matrices T represent Cartesian poses in SE(3) format. Arrows indicate the flow and transformation of data between processing steps.

The framework assumes that the simulated robotic tool executes the joint configuration generated by the IK solver without additional low‐level joint dynamics. Under this assumption, the real2sim kernel can operate independently of the simulation platform without requiring feedback of the simulated robotic tool's joint states. Consequently, given an appropriate Cartesian pose trajectory from the real robot, the corresponding joint configurations of the simulated robotic tool can be calculated sequentially by the real2sim kernel.

The updated joint states generated by the numerical IK solver are subsequently published to a ROS topic and can be subscribed to by external simulation or visualisation platforms, such as Unity, Blender, MATLAB, V‐REP/CoppeliaSim, and Gazebo. In this work, Unity is used as the primary simulation and visualisation platform because its PhysX engine provides physics‐based simulation capabilities required for the simulated tissue‐manipulation tasks. The simulated robotic tools are visualised in Unity, where tissue‐grasping and cutting tasks are performed.

### 2.6 Soft Tissue Simulation

The framework is intended to support the evaluation of robotic tool designs through simulated surgical tasks, such as tissue cutting, suturing, and knot tying. In this work, a simulated tissue‐manipulation task involving grasping and cutting is developed to evaluate the proposed framework. Specifically, one simulated robotic tool is used as a grasper to grasp the tissue, while the other is used as scissors to perform the cutting action.

To implement the soft tissue simulation, the PhysX 5 SDK for Unity is employed. PhysX 5 provides physics‐based simulation capabilities including the finite element method (FEM) for soft bodies, position‐based dynamics (PBD), and fluid simulation [34].

To provide sufficient spatial resolution for tissue deformation and cutting, the simulated soft tissue was modelled as a planar mesh consisting of 2, 601 vertices and 5, 000 triangles. The relatively dense mesh allows localised deformation and geometric changes associated with tool‐tissue interaction and cutting to be represented with greater spatial detail.

Haptic feedback was rendered during tool‐tissue interaction based on the penetration‐induced deformation of the simulated tissue. A linear spring model was employed, in which the rendered force is proportional to the penetration depth and directed opposite to the tool penetration. The force‐rendering model is expressed as

(13) $$F_{\mathrm{haptic}}=k_{stiff}\left(P_{tissue}-P_{tool}\right)$$

where $k_{stiff}$ is the stiffness coefficient of the simulated tissue, with $k_{stiff}=0.05$ N/mm used in this work. $P_{\mathrm{tissue}}$ denotes the position of the tissue surface at the tool‐tissue contact location, and $P_{\mathrm{tool}}$ denotes the corresponding position of the interacting point on the simulated robotic tool. Therefore, the vector $\left(P_{tissue}-P_{tool}\right)$ represents the penetration‐induced deformation vector, whose magnitude corresponds to the penetration depth and whose direction determines the direction of the restoring force. The resulting force $F_{\mathrm{haptic}}$ is rendered to the user through the haptic device. In the current implementation, frictional forces at the tool‐tissue interface are not included in the haptic rendering. The haptic force commands were transmitted to the haptic devices at 500 Hz, while the Unity physics simulation used a fixed timestep of 0.02 s, corresponding to a nominal physics update rate of 50 Hz.

### 2.7 Robotic Systems

To establish a portable real2sim bimanual teleoperation system based on the proposed framework, two 6‐DOF (RRRRRR) Touch devices (formerly Phantom Omni; 3D Systems Inc., Cary, NC, USA) were used as Real Robots A1 and A2 in *Experiment 1*. In *Experiment 2*, the two Touch devices were replaced by two master tool manipulators (MTMs) from the da Vinci Research Kit (dVRK), which served as Real Robots A1 and A2. In both experiments, two newly designed 6‐DOF (PRRRRR) robotic tools (a grasper and scissors developed for cleft palate surgery, as illustrated in Figure 1) were used as Sim Tools B1 and B2. The simulated environment and robotic tools were implemented in Unity (version 2022.3.34f1, Unity Technologies, San Francisco, CA, USA).

*Experiments 3–6* were based on the Omni‐to‐Unity configuration established in Experiment 1, with modifications according to their respective evaluation or demonstration objectives. *Experiment 3* used a single Omni device to simultaneously teleoperate two versions of a simulated robotic tool for comparison of their kinematic behaviour. *Experiment 4* retained the bimanual configuration but replaced the right‐hand 6‐DOF simulated robotic tool with an 11‐DOF simulated robotic system and additionally incorporated haptic feedback. *Experiments 5 and 6* were conducted as supplementary demonstrations: Experiment 5 used two simulated 7‐DOF Franka robots as followers, whereas Experiment 6 used a physical 7‐DOF Franka robot as the follower to demonstrate a real2real implementation. The robotic systems and objectives of Experiments 1–6 are summarised in Table 2.

**TABLE 2:** Overview of the robotic systems and evaluation goals in the experiments.

	System	Set ^a^	Leader	Follower	Evaluation Goal
Exp.1	Omni‐to‐Unity	L,R	6‐DOF Omni (x2)	6‐DOF tool (x2)	Evaluation of Omni‐to‐Unity bimanual teleoperation
Exp.2	dVRK‐to‐Unity	L,R	7‐DOF MTM (x2)	6‐DOF tool (x2)	Evaluation with a different leader robot
Exp.3	Omni‐to‐Unity	R	6‐DOF Omni (x1)	6‐DOF tool (x2)	Application to robotic tool design
Exp.4	Omni‐to‐Unity	L,R	6‐DOF Omni (x2)	6‐DOF tool (x1); 11‐DOF tool (x1)	Evaluation with different follower kinematics and haptic feedback
Exp.5	Omni‐to‐Panda	L,R	6‐DOF Omni (x2)	7‐DOF Franka robot (x2)	Supplementary demonstration with redundant follower robots
Exp.6	Omni‐to‐Panda	R	6‐DOF Omni (x1)	7‐DOF Franka robot (x1)	Supplementary demonstration of real2real teleoperation

*Note:* Exp.1‐Exp.6 denote Experiments 1–6, respectively.

^a^ L and R denote the left‐ and right‐hand teleoperation systems, respectively.

The kinematics of the Omni device are described in [35]. For the Omni devices, a ROS catkin workspace was used to publish their real‐time EE Cartesian poses at 1000 Hz. A separate catkin workspace contained the real2sim kernel implemented in C++, which processed the leader‐robot data and generated the corresponding joint states of the simulated robotic tools. For the dVRK MTMs, Cartesian pose data were available through the existing ROS interface at 100 Hz; therefore, only the real2sim kernel was required for integration. The real2sim kernel operated at 500 Hz in Experiments 1 and 3–6 and at 100 Hz in Experiment 2. The resulting joint states of the simulated robotic tools were published through ROS and subscribed to by Unity for real‐time visualisation and interaction.

The Omni‐based systems used in Experiments 1 and 3–6 were implemented on a computer equipped with a 13th Gen Intel Core i5‐13600K × 20 processor, 31.1 GiB of memory, and Ubuntu 20.04.6 LTS 64‐bit operating system. The dVRK‐to‐Unity system used in Experiment 2 was implemented on a computer with the same memory and operating system but equipped with an Intel Core i5‐14600K × 20 processor. A GOOVIS G3 MAX 3D Head Mounted Display (Shenzhen NED Optics Co. Ltd., China) was used for stereoscopic visualisation of the Unity environment. The Omni‐to‐Unity and dVRK‐to‐Unity experimental setups are shown in Figure 3.

**FIGURE 3:**
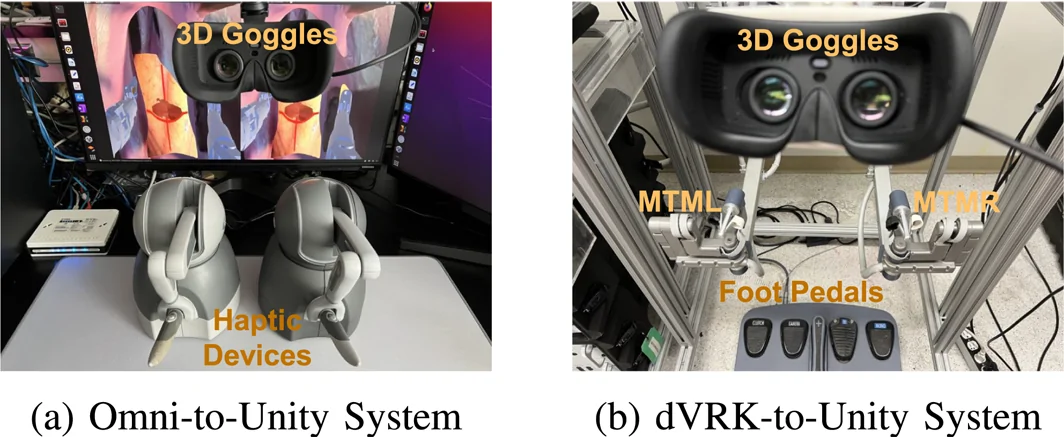
Experimental setups.

### 2.8 Parameterisation

A series of experiments was conducted to evaluate the implemented real2sim bimanual teleoperation systems and to examine the framework under different leader and follower configurations. Experiments 1 and 2 evaluated the two primary bimanual teleoperation systems using the same simulated tissue grasping‐and‐cutting task. Experiments 3 and 4 examined an application to robotic tool design and a configuration with different follower kinematics, respectively. Experiments 5 and 6 were included as supplementary demonstrations of additional framework capabilities.

In Experiment 1, the Omni‐to‐Unity system was evaluated during a simulated bimanual tissue grasping‐and‐cutting task. One simulated robotic tool was teleoperated to grasp and hold the tissue while the other was teleoperated to perform the cutting motion. During the task, the desired Cartesian poses, numerical IK solutions, Cartesian pose errors of the IK solutions, and IK computation times were recorded to characterise the behaviour of the implemented teleoperation framework. In Experiment 2, the same simulated robotic tools and task procedure were used, while the two Omni devices were replaced by the dVRK MTMs. This configuration was used to evaluate the implementation of the framework with a different type of leader robot.

Experiment 3 was conducted as an application demonstration for robotic tool design. Two versions of the same simulated robotic tool, differing in the length of the fourth link, were modelled using their respective DH parameters and teleoperated simultaneously using the same Omni input. Their motions were visualised with either their bases or distal joints aligned, allowing their kinematic behaviour under the same leader input to be directly compared. This experiment was intended to illustrate how the framework can be used to support qualitative comparison between different iterations of a robotic tool design.

In Experiment 4, the framework was evaluated with a follower system having different kinematic characteristics from those used in Experiments 1 and 2. Based on the Omni‐to‐Unity bimanual configuration, the left‐hand 6‐DOF simulated robotic tool was retained, while the right‐hand 6‐DOF tool was replaced by an 11‐DOF simulated robotic system consisting of a 7‐DOF Franka Emika Research 3 robot coupled with the last four joints of the robotic scissors. The user performed a bimanual tissue grasping‐and‐cutting task similar to that in Experiment 1. Haptic feedback associated with tool‐tissue interaction was additionally rendered in Unity and transmitted to the Omni devices. The joint trajectories of the two follower systems and the rendered force‐feedback signals were recorded to examine the framework operation with the different follower kinematics and the integration of haptic feedback.

Experiments 5 and 6 were conducted as supplementary demonstrations rather than as quantitative evaluations. In Experiment 5, the 6‐DOF simulated robotic tools were replaced by two simulated 7‐DOF Franka robots to demonstrate implementation with kinematically redundant follower robots. In Experiment 6, the right‐hand Omni‐to‐Panda configuration was implemented using a physical 7‐DOF Franka robot under joint position control, providing a preliminary demonstration of extending the framework from real2sim to real2real teleoperation.

The principal configuration parameters used in Experiments 1–6 are summarised in Table 3, including the initial joint configurations of the follower systems, leader‐to‐follower transformation matrices, motion scaling factors, and relevant tool dimensions. Additional parameters associated with the individual system components are provided in the corresponding methodological descriptions. A demonstration video covering Experiments 1–6 is provided as the accompanying Video 1 and is also available online (https://youtu.be/DSaiMpGy6e8).

**TABLE 3:** Configuration parameters for the teleoperation systems used in the experiments.

	Left‐hand teleoperation system				Right‐hand teleoperation system			
	$q_{B.init}$	TAB	s	Length (m)	$q_{B.init}$	TAB	s	Length (m)
	(Alg. 1)	(Eqn. (3))	(Eqn. (4))	(Table 1)	(Alg. 1)	(Eqn. (3))	(Eqn. (4))	(Table 1)
Exp.1	$\left[\begin{array}{c} 0\\ 0\\ 0\\ 10{}^{\circ}\\ 10{}^{\circ}\\ 0 \end{array}\right]$	$\left[\begin{array}{cccc} 0 & 1 & 0 & 0\\ 0 & 0 & -1 & 0\\ -1 & 0 & 0 & 0\\ 0 & 0 & 0 & 1 \end{array}\right]$	0.0001	$L_{2}=0.24175$ $L_{3}=0.0064$ $L_{4}=0.0235$ $L_{5}=0.00454$	$\left[\begin{array}{c} 0\\ 0\\ 0\\ -10{}^{\circ}\\ 10{}^{\circ}\\ 0 \end{array}\right]$	$\left[\begin{array}{cccc} 0 & 1 & 0 & 0\\ 0 & 0 & -1 & 0\\ -1 & 0 & 0 & 0\\ 0 & 0 & 0 & 1 \end{array}\right]$	0.0001	$L_{2}=0.24175$ $L_{3}=0.0064$ $L_{4}=0.0235$ $L_{5}=0.00454$
Exp.2	$\left[\begin{array}{c} 0\\ 0\\ 0\\ 10{}^{\circ}\\ 10{}^{\circ}\\ 0 \end{array}\right]$	$\left[\begin{array}{cccc} 0 & 1 & 0 & 0\\ 1 & 0 & 0 & 0\\ 0 & 0 & -1 & 0\\ 0 & 0 & 0 & 1 \end{array}\right]$	0.2	$L_{2}=0.24175$ $L_{3}=0.0064$ $L_{4}=0.0235$ $L_{5}=0.00454$	$\left[\begin{array}{c} 0\\ 0\\ 0\\ -10{}^{\circ}\\ 10{}^{\circ}\\ 0 \end{array}\right]$	$\left[\begin{array}{cccc} 0 & 1 & 0 & 0\\ 1 & 0 & 0 & 0\\ 0 & 0 & -1 & 0\\ 0 & 0 & 0 & 1 \end{array}\right]$	0.2	$L_{2}=0.24175$ $L_{3}=0.0064$ $L_{4}=0.0235$ $L_{5}=0.00454$
Exp.3	NA	NA	NA	NA	$\left[\begin{array}{c} 0\\ 0\\ 0\\ 0\\ 0\\ 0 \end{array}\right]$	$\left[\begin{array}{cccc} 0 & 1 & 0 & 0\\ 0 & 0 & -1 & 0\\ -1 & 0 & 0 & 0\\ 0 & 0 & 0 & 1 \end{array}\right]$	0.0001	$L_{2}=0.24175$ $L_{3}=0.0064$ $L_{4,tool1}=0.0235$ $L_{4,tool2}=0.0335$ $L_{5}=0.00454$
Exp.4	$\left[\begin{array}{c} 0\\ 0\\ 0\\ 10{}^{\circ}\\ 10{}^{\circ}\\ 0 \end{array}\right]$	$\left[\begin{array}{cccc} 0 & 1 & 0 & 0\\ 0 & 0 & -1 & 0\\ -1 & 0 & 0 & 0\\ 0 & 0 & 0 & 1 \end{array}\right]$	0.0001	$L_{2}=0.24175$ $L_{3}=0.0064$ $L_{4}=0.0235$ $L_{5}=0.00454$	$\left[\begin{array}{c} 0\\ -45{}^{\circ}\\ 0\\ -135{}^{\circ}\\ 0\\ 90{}^{\circ}\\ 45{}^{\circ}\\ 0\\ 0\\ 0\\ 0 \end{array}\right]$ ^a^	$\left[\begin{array}{cccc} -1 & 0 & 0 & 0\\ 0 & 0 & 1 & 0\\ 0 & 1 & 0 & 0\\ 0 & 0 & 0 & 1 \end{array}\right]$	0.0001	$L_{2}=0.2$ $L_{3}=0.0064$ $L_{4}=0.0235$ $L_{5}=0.00454$
Exp.5	$\left[\begin{array}{c} 0\\ -45{}^{\circ}\\ 0\\ -135{}^{\circ}\\ 0\\ 90{}^{\circ}\\ 45{}^{\circ} \end{array}\right]$	$\left[\begin{array}{cccc} 1 & 0 & 0 & 0\\ 0 & 0 & 1 & 0\\ 0 & -1 & 0 & 0\\ 0 & 0 & 0 & 1 \end{array}\right]$	0.0005	NA	$\left[\begin{array}{c} 0\\ -45{}^{\circ}\\ 0\\ -135{}^{\circ}\\ 0\\ 90{}^{\circ}\\ 45{}^{\circ} \end{array}\right]$	$\left[\begin{array}{cccc} -1 & 0 & 0 & 0\\ 0 & 0 & 1 & 0\\ 0 & 1 & 0 & 0\\ 0 & 0 & 0 & 1 \end{array}\right]$	0.0005	NA
Exp.6	NA	NA	NA	NA	$\left[\begin{array}{c} 0\\ -45{}^{\circ}\\ 0\\ -135{}^{\circ}\\ 0\\ 90{}^{\circ}\\ 45{}^{\circ} \end{array}\right]$	$\left[\begin{array}{cccc} -1 & 0 & 0 & 0\\ 0 & 0 & 1 & 0\\ 0 & 1 & 0 & 0\\ 0 & 0 & 0 & 1 \end{array}\right]$	0.0005	NA

*Note:* Exp.1‐Exp.6 denote Experiments 1–6, respectively.

Abbreviation: NA, not applicable.

^a^ The first seven elements correspond to the joints of the Franka robot, while the last four elements correspond to the last four joints of the robotic tool shown in Table 1.

## 3 Results

In this section, results from the experiments described earlier are presented. *Experiments 1 and 2* evaluate the two bimanual teleoperation systems established using the proposed framework, while *Experiments 3 and 4* examine its application to robotic tool design and its use with a robotic tool having different kinematic characteristics, respectively. *Experiments 5 and 6* are presented as supplementary demonstrations of additional framework capabilities.

### 3.1 Evaluation of Bimanual Teleoperation Systems

This subsection presents the results obtained using the two bimanual teleoperation systems established according to the proposed framework. Experiment 1 evaluates the portable Omni‐to‐Unity system, whereas Experiment 2 evaluates the dVRK‐to‐Unity system using the same simulated robotic tools and a similar bimanual task.

In *Experiment 1*, two Omni haptic devices (Real Robots A1 and A2) were used to teleoperate two simulated robotic tools (Sim Tools B1 and B2) during a virtual bimanual tissue‐manipulation task. Specifically, Real Robot A1 controlled Sim Tool B1 for tissue grasping, while Real Robot A2 controlled Sim Tool B2 for tissue cutting (see the accompanying Video 1). An example of the resulting tissue cutting is shown in Figure 4a. During the task, the desired Cartesian poses, numerical IK solutions, IK pose errors, and IK computation times were recorded to characterise the behaviour of the implemented real2sim teleoperation framework.

**Video 1:** Demonstration of the experiments conducted using the proposed Real2Sim bimanual teleoperation framework.

**FIGURE 4:**
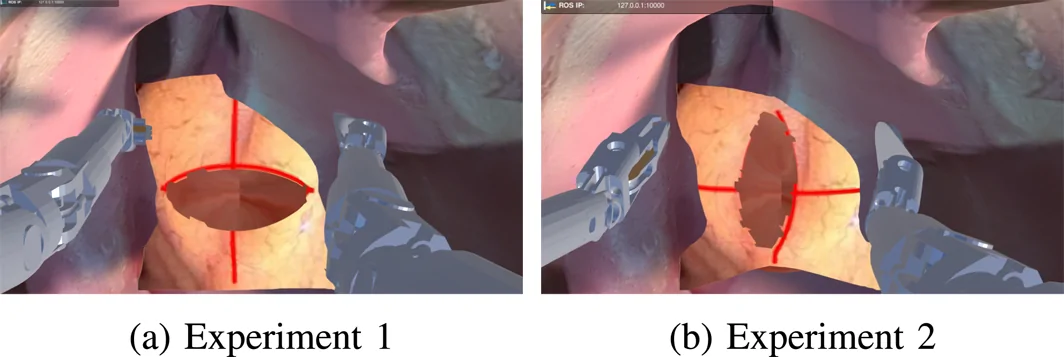
Examples of simulated tissue cutting results obtained in Experiments 1 and 2 within an infant mouth model.

In Experiment 1, the desired Cartesian poses of the simulated robotic tools are shown in Figure 5, and the corresponding 3D position trajectories are shown in Figure 6a and 6b. The two Omni devices (Real Robots A1 and A2) have separate but consistently defined base coordinate systems. For visualisation, the trajectories corresponding to both devices are plotted within the same coordinate representation in Figure 6.

**FIGURE 5:**
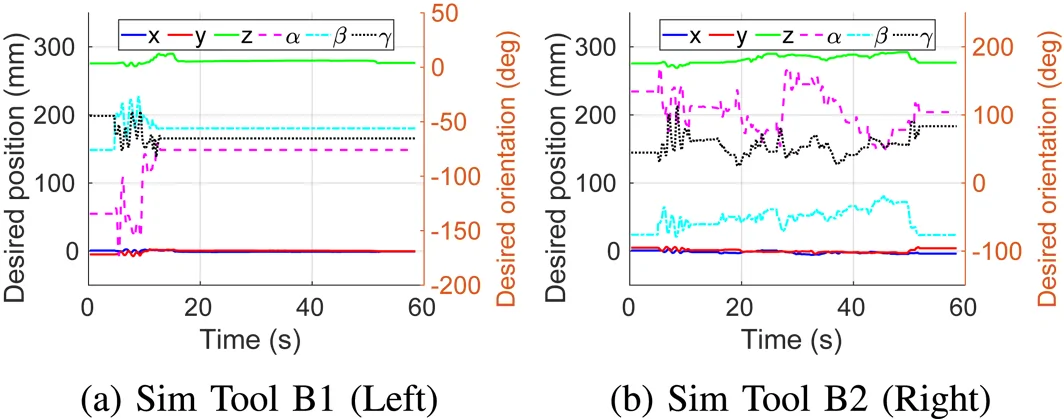
Desired Cartesian poses of the simulated robotic tools during Experiment 1. The 3D position is represented by [x,y,z], and the 3D orientation is represented by the roll‐pitch‐yaw Euler angles [α,β,γ].

**FIGURE 6:**
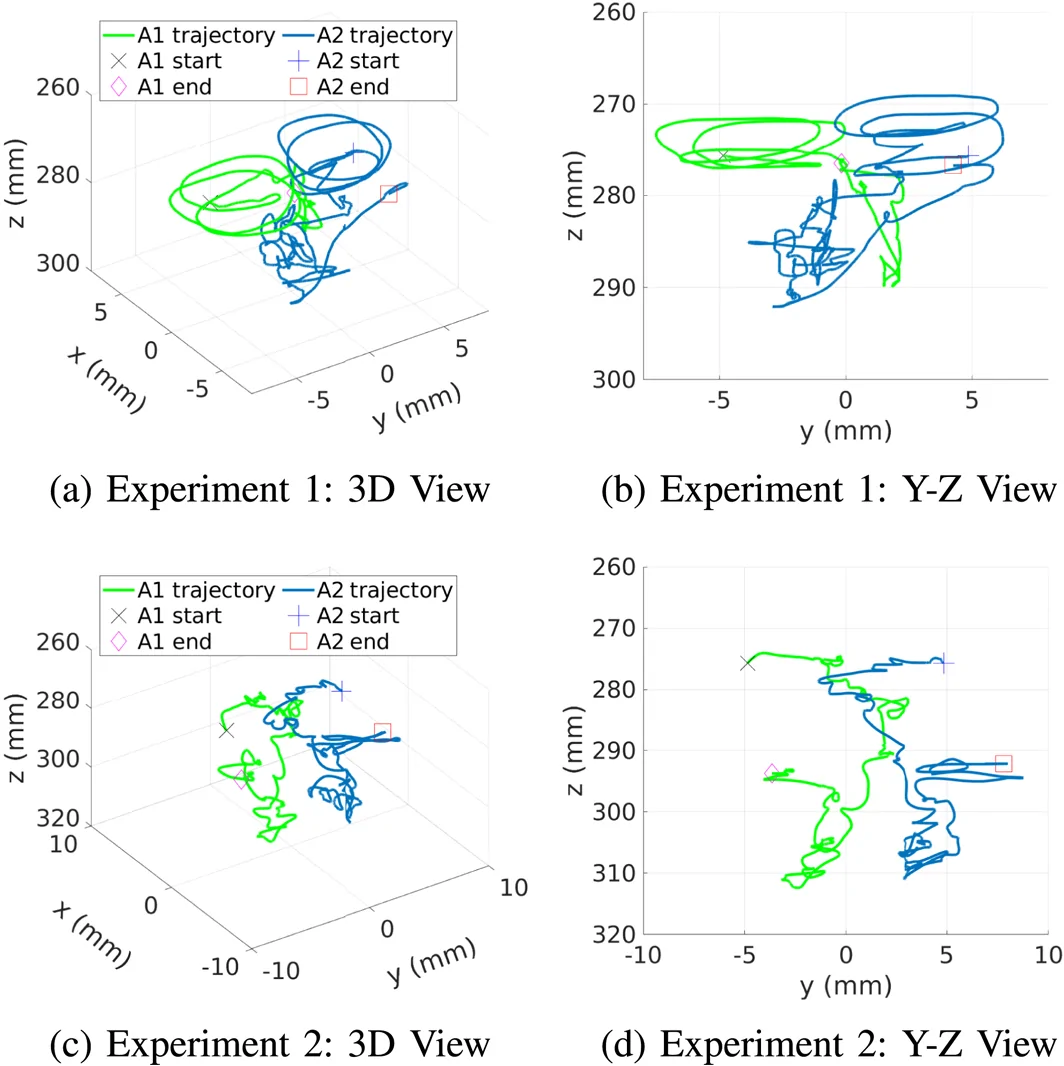
Desired position trajectories of the simulated robotic tools.

The numerical IK solutions corresponding to the desired Cartesian poses are shown in Figure 7. For the reported task, continuous joint trajectories were obtained for both simulated robotic tools throughout the teleoperated motions. As shown in Figure 7a, the joint configuration of Sim Tool B1 remained nearly unchanged during approximately 15∼50 s, apart from small variations. During this interval, Sim Tool B1 was used to grasp and hold the simulated tissue while Sim Tool B2 performed the cutting motion.

**FIGURE 7:**
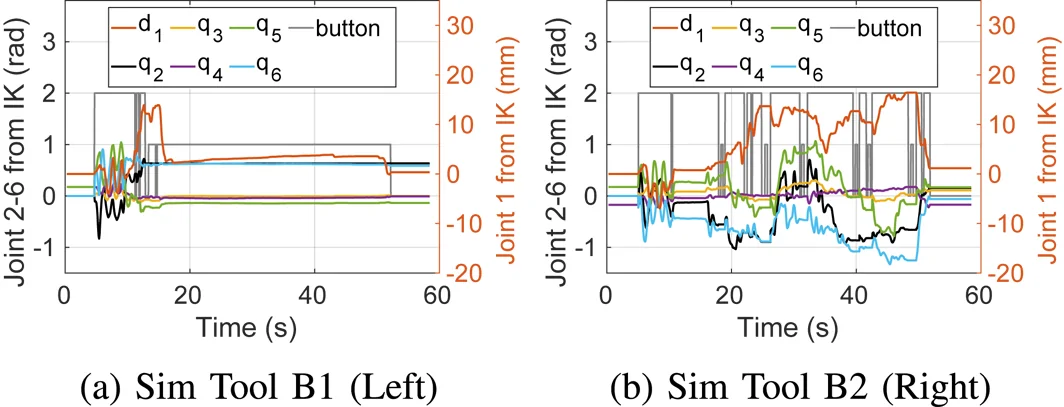
Joint trajectories generated by the numerical IK solver during the bimanual tissue grasping‐and‐cutting task in Experiment 1. The button state indicates the teleoperation mode: button=0, teleoperation disengaged; button=1, 3D position control with tool‐jaw actuation; and button=2, full 6D pose control.

The button states shown in Figure 7 indicate the teleoperation modes used in Experiment 1. When button=0, teleoperation is disengaged. When button=1, only 3D position mapping is enabled while the orientation of the simulated robotic tools remains unchanged. This mode is also used to control opening and closing of the tool jaws. When button=2, full 6D pose mapping is enabled. Both buttons additionally provide a clutching function to accommodate the difference in workspace size between the Omni devices and the simulated robotic tools. These button assignments are specific to the Omni device and can be adapted to the input mechanisms available on other leader robots. For example, the dVRK MTMs use finger grips and foot pedals, as implemented in Experiment 2.

The Cartesian poses corresponding to the numerical IK solutions were reconstructed using the FK of the simulated robotic tools and compared with the desired poses shown in Figure 5. The resulting Cartesian pose errors are presented in Figure 8. During the reported task, the position and orientation errors remained below approximately $5.5\times 10^{-4}$ mm and $5.2\times 10^{-4}$ deg in absolute magnitude, respectively. The maximum absolute position and orientation errors were $5.3887\times 10^{-4}$ mm and $5.0862\times 10^{-4}$ deg, respectively, for Sim Tool B1 (left), and $5.4803\times 10^{-4}$ mm and $5.1863\times 10^{-4}$ deg, respectively, for Sim Tool B2 (right). These results indicate that the numerical IK solutions closely tracked the desired Cartesian poses for the trajectories evaluated in Experiment 1.

**FIGURE 8:**
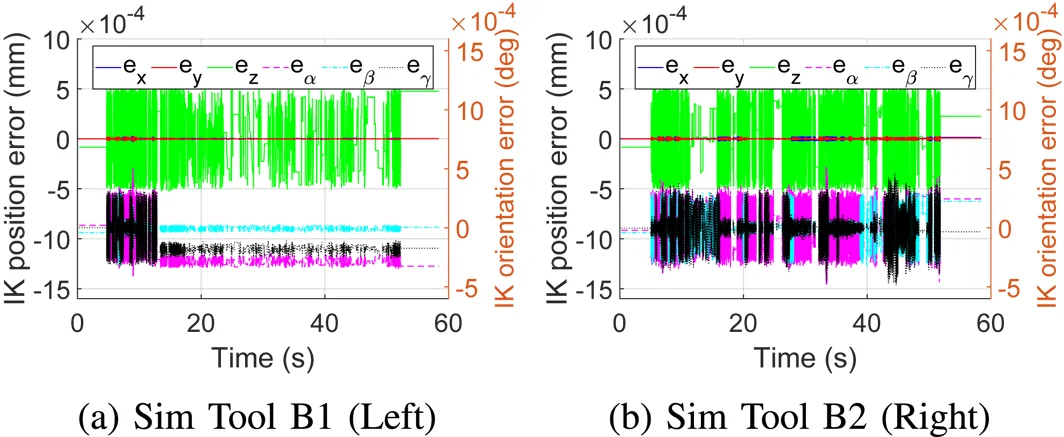
Cartesian pose errors of the numerical IK solutions during Experiment 1.

The computation time of the IK solver affects the control rate that can be supported by the real2sim framework. The IK computation times recorded during Experiment 1 are shown in Figure 9, with summary statistics provided in Table 4. For the reported task, most IK calculations were completed within 3 ms. The mean computation times considering all nonzero values were 0.5046 and 1.3452 ms for Sim Tools B1 and B2, respectively. The comparatively low mean value for Sim Tool B1 reflects the interval during which the grasper remained nearly stationary while holding the tissue, resulting in low but nonzero IK computation times. To provide additional characterisation of the active IK calculations, the mean and standard deviation for computation times greater than 1 ms are also reported in Table 4.

**FIGURE 9:**
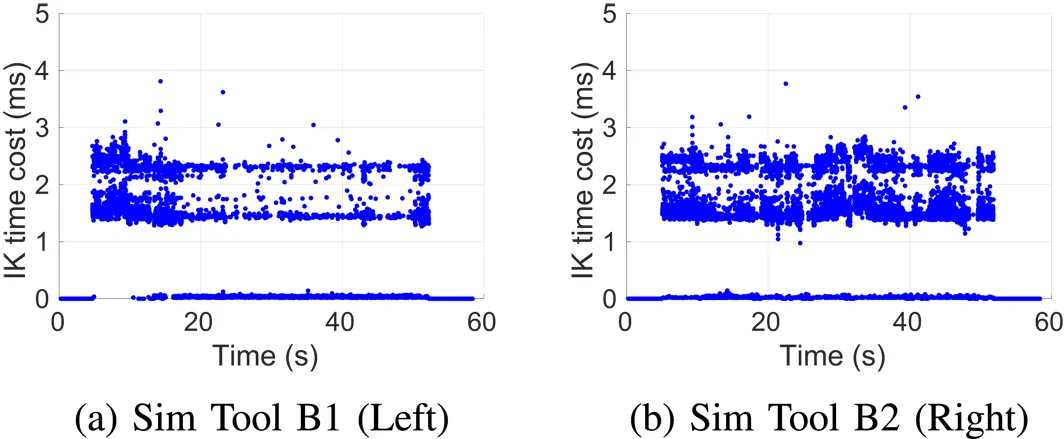
IK computation times recorded during Experiment 1.

**TABLE 4:** IK computation time statistics for Experiments 1 and 2.

		Sim Tool B1 (Left)					Sim Tool B2 (Right)				
	Included samples	Mean	std.	p95	p99	Max	Mean	std.	p95	p99	Max
Exp.1	>0 ms	0.5046	0.8010	2.2967	2.4815	3.8109	1.3452	0.7475	2.4419	2.6004	3.7682
Exp.1	>1 ms	1.7715	0.4064	2.4498	2.6329	3.8109	1.6886	0.3676	2.4687	2.6231	3.7682
Exp.2	>0 ms	1.7944	0.6258	2.5630	2.8685	9.5332	1.8666	0.4756	2.4280	3.0476	7.3338
Exp.2	>1 ms	1.8913	0.4842	2.5682	2.8777	9.5332	1.8724	0.4651	2.4283	3.0501	7.3338

*Note:* Computation times are reported in milliseconds. ‘>0 ms’ includes all nonzero IK computation time samples, whereas ‘>1 ms’ includes only samples with computation times greater than 1 ms. p95 and p99 denote the 95th and 99th percentiles, respectively. Max denotes the maximum computation time among the included samples. Exp.1, Experiment 1; Exp.2, Experiment 2; std., standard deviation.

In *Experiment 2*, the two Omni devices were replaced by the two MTMs of the dVRK while retaining the simulated robotic tools used in Experiment 1. The same type of simulated tissue grasping‐and‐cutting task was performed to examine whether the framework could be configured with a different leader robot. An example of the resulting tissue cutting is shown in Figure 4b. In this implementation, the MTM finger grips were used to control opening and closing of the simulated tool jaws.

The desired 3D position trajectories obtained in Experiment 2 are shown in Figure 6c and 6d. Similar to the Omni devices in Experiment 1, the two MTMs have separate but consistently defined base coordinate systems. For visualisation, the trajectories corresponding to the two MTMs are plotted within the same coordinate representation.

The corresponding numerical IK solutions obtained in Experiment 2 are shown in Figure 10. Continuous joint trajectories were obtained for both simulated robotic tools during the reported bimanual tissue grasping‐and‐cutting task. The button states shown in Figure 10 correspond to the state of the dVRK foot pedal: button=0 indicates that teleoperation is disengaged, whereas button=1 indicates that 6D pose teleoperation is enabled. Opening and closing of the simulated tool jaws were controlled separately using the MTM finger grips and are therefore not represented by the button state in the figure.

**FIGURE 10:**
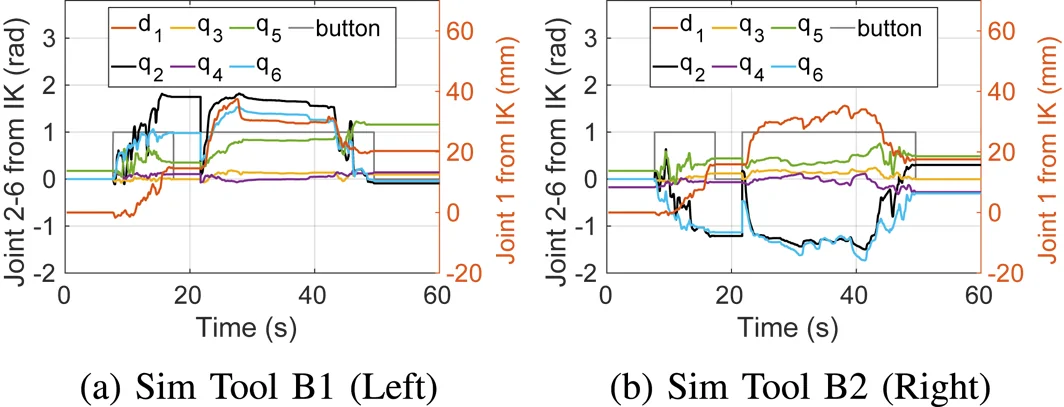
Joint trajectories obtained from the numerical IK solver during Experiment 2. The button state indicates the teleoperation mode: button=0, teleoperation disengaged (foot pedal released), and button=1, full 6D pose control enabled (foot pedal pressed). Grasping and cutting actions were controlled separately using the dVRK MTM finger grips and are therefore not represented by the button state.

The IK computation times recorded during Experiment 2 are shown in Figure 11, with the corresponding summary statistics for Experiments 1 and 2 reported in Table 4. Considering all nonzero samples, the mean computation times in Experiment 2 were 1.7944 and 1.8666 ms for Sim Tools B1 and B2, respectively, with p95 values of 2.5630 and 2.4280 ms and p99 values of 2.8685 and 3.0476 ms. The maximum observed computation times were 9.5332 and 7.3338 ms, respectively, both below the 10‐ms processing period used in Experiment 2. For comparison, the p99 computation times in Experiment 1 were 2.4815 and 2.6004 ms, with maximum values of 3.8109 and 3.7682 ms for Sim Tools B1 and B2, respectively. Overall, across the reported tasks in both experiments, the mean computation times ranged from 0.5046 to 1.8666 ms, while the p99 values ranged from 2.4815 to 3.0476 ms. These measurements provide a quantitative characterisation of both the typical and upper‐tail IK computation times observed during the evaluated teleoperation tasks.

**FIGURE 11:**
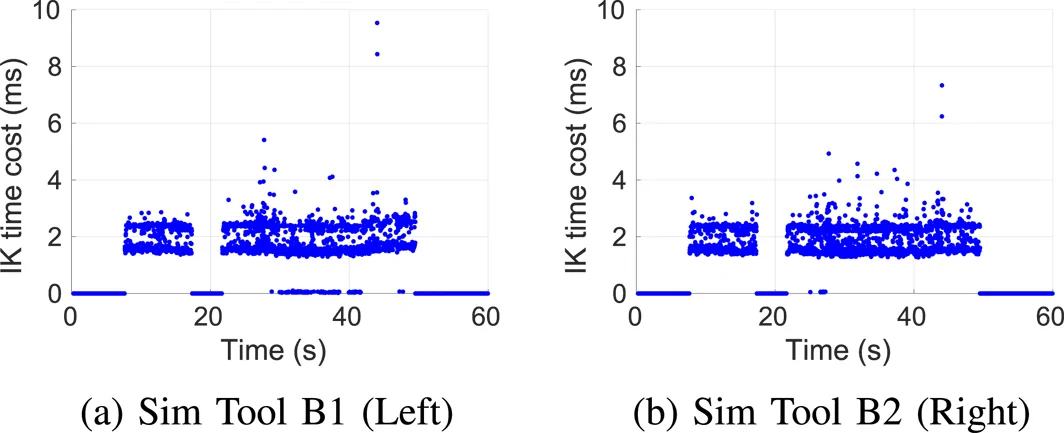
IK computation times recorded during Experiment 2.

Across the reported trials in Experiments 1 and 2, the IK solver generated continuous numerical solutions without visually apparent discontinuities in the recorded joint trajectories (Figures 7 and 10). Together with the measured Cartesian pose errors and IK computation times, these results provide empirical evidence that the implemented IK solver supported continuous real‐time teleoperation for the simulated tasks and system configurations evaluated in these experiments.

### 3.2 Application to Robotic Tool Design

During robotic tool development, different design versions can be evaluated using analyses such as reachability and force characterisation [7, 36]. In addition to these quantitative analyses, simultaneous visualisation of different tool designs under the same teleoperation input can provide complementary information regarding their kinematic behaviour. Figure 12 illustrates such an application using the proposed framework.

**FIGURE 12:**
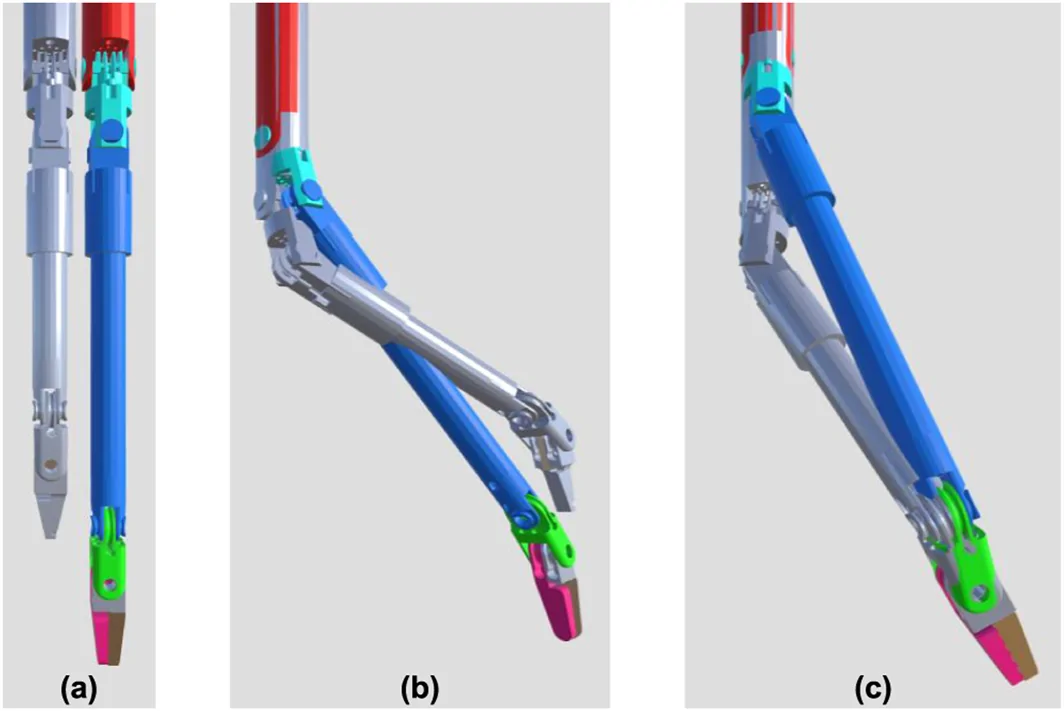
Application of the proposed framework to robotic tool design. (a) Two versions of the robotic tool: the original design (silver) and a modified design (coloured) with an increased fourth‐link length. (b) Comparison with the two tool models aligned at the same base. (c) Comparison with the two tool models aligned at the same distal joint. For clarity, the first link and most of the second link are omitted from the visualisation.

Figure 12a shows two versions of a robotic tool design: the original design (silver) and a modified design (coloured) with an increased length of the fourth link (blue). Two visualisation configurations were considered: aligning the two tools at the same base (Figure 12b) and aligning them at the same distal joint (Figure 12c). In *Experiment 3*, both tool models were teleoperated simultaneously using the same haptic‐device input, while their respective kinematics were modelled using separate DH parameters within the framework. This configuration allowed the motion of the two designs to be directly compared under the same leader input. In the example shown in Figure 12 and the accompanying video, the original design exhibited a larger displacement of the elbow region than the modified design for the demonstrated motion. This experiment illustrates how the framework can be used to visually compare the kinematic behaviour of different tool designs. Quantitative comparison of design performance would require additional task‐specific metrics and repeated evaluations and is left for future work.

The framework has also been used in subsequent work involving robotic tool design, workspace and dexterity analyses, and real‐to‐real teleoperation evaluation [37], providing an additional application example beyond the experiments reported here.

### 3.3 Evaluation With Different Follower Kinematics and Haptic Feedback

To examine the framework with a simulated robotic tool having different kinematic characteristics, *Experiment 4* was conducted using the Omni‐to‐Unity bimanual teleoperation system established in Experiment 1. The right‐hand 6‐DOF simulated robotic tool was replaced by an 11‐DOF simulated robotic system, while the left‐hand 6‐DOF tool was retained. The 11‐DOF system was formed by coupling the last four joints of Sim Tool B2 (the scissors illustrated in Figure 1) to the flange of a 7‐DOF Franka Emika Research 3 virtual robot, resulting in a follower system with different kinematics and kinematic redundancy relative to the 6‐DOF tool.

The 11‐DOF Sim Tool B2 was incorporated into the framework by specifying the configuration parameters listed in Table 5, including its DH parameters, initial joint configuration, leader‐to‐follower transformation matrix, and motion scaling factor. Joint limits, the joint‐state publisher, and the URDF model were additionally configured as required for the implementation and visualisation. In this example, modification of these parameters was sufficient to configure the framework for the 11‐DOF follower, providing an experimental example of its use with a kinematically different simulated robotic system.

**TABLE 5:** Configuration parameters for integrating the 11‐DOF Sim Tool B2 into the framework.

Necessity	Parameter	Description
Required	${dh}_{B}$	DH parameters of Sim Tool B2
Required	$q_{B.init}$	Initial joint configuration of Sim Tool B2
Required	TAB	Transformation matrix from A2 to B2
Required	s	Motion scaling factor from A2 to B2
Optional	Joint limits	Joint limits of Sim Tool B2
Optional	Publisher of $q_{B.now}$	Joint‐state publisher for visualisation
Optional	URDF	Sim Tool B2's URDF for visualisation

*Note:* In the Parameter column, ‘B2’ is abbreviated as ‘B’ for consistency.

Abbreviation: URDF, unified robot description format.

During Experiment 4, the two simulated robotic tools were teleoperated bimanually using a pair of Omni haptic devices. A tissue‐manipulation sequence similar to Experiment 1 was performed: the tools were first moved towards the simulated tissue, the left‐hand grasper was then used to grasp the tissue, and the right‐hand scissors were subsequently used to perform the cutting motion.

Haptic feedback generated from interactions between the simulated robotic tools and tissue was rendered in Unity and transmitted to the Omni devices. The recorded force‐feedback signals are presented together with the corresponding simulated robotic tool joint trajectories in Figure 13.

**FIGURE 13:**
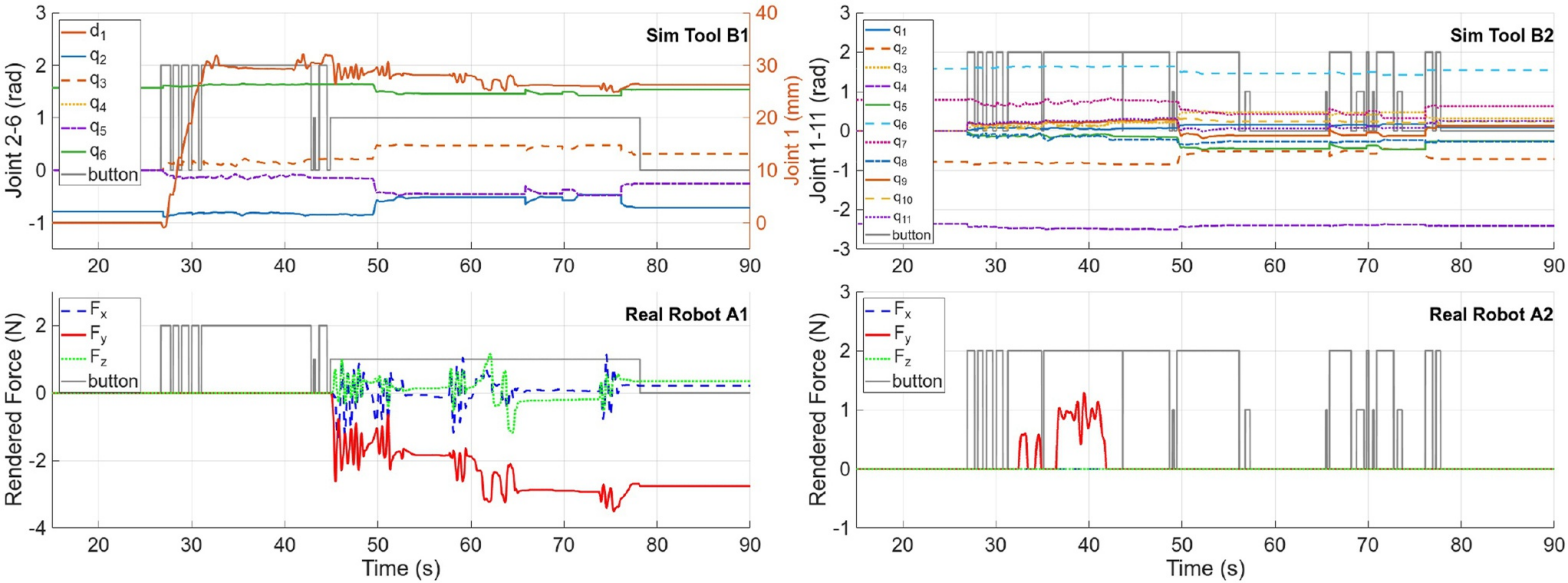
Joint trajectories and rendered haptic feedback recorded during Experiment 4 with different follower kinematics. Sim Tool B1 (left‐hand side) has 6 DOFs, whereas Sim Tool B2 (right‐hand side) has 11 DOFs. The button state indicates the teleoperation mode: button=0, teleoperation disengaged; button=1, 3D position control with tool‐jaw actuation; and button=2, full 6D pose control.

The results from Experiment 4 are shown in Figure 13, with the left two plots corresponding to the left‐hand teleoperation system and the right two plots corresponding to the right‐hand system. For interpretation of the recorded sequence, approximately 27∼32 s corresponds to motion towards the tissue, 32∼44 s to tissue probing, and 44∼78 s to tissue grasping and cutting. The upper plots show the joint trajectories generated by the IK solver for the 6‐DOF and 11‐DOF simulated robotic systems, respectively, while the lower plots show the corresponding rendered force‐feedback signals. Nonzero force feedback signals were observed during tool‐tissue interaction: for Sim Tool B1 during tissue grasping and subsequent small motions of the grasped tissue, and for Sim Tool B2 during tissue probing. These results confirm that haptic signals were generated and transmitted during the specified interaction periods in the reported task.

### 3.4 Supplementary Demonstrations

Experiment 4 provides an example of applying the numerical IK solver to an 11‐DOF simulated robotic system with kinematic redundancy. As an additional demonstration, *Experiment 5* in the accompanying video implements a real2sim Omni‐to‐Panda bimanual teleoperation system (A demo code for real2sim Omni‐to‐Panda bimanual teleoperation is available at https://github.com/teng4/Real2Sim‐Omni‐to‐Panda‐Bimanual‐Teleoperation), in which two simulated 7‐DOF Franka robots are teleoperated using two Omni devices. Because each Franka robot has seven joints for a 6‐DOF Cartesian pose task, this demonstration provides an additional example of applying the framework to a kinematically redundant follower system.

Although the framework was developed primarily for real2sim teleoperation, its joint‐command output can also be applied to a physical follower robot when an appropriate low‐level control interface is available. In supplementary *Experiment 6*, the right‐hand Omni‐to‐Panda configuration from Experiment 5 was implemented with a physical 7‐DOF Franka Emika Research 3 using joint‐position control. This demonstration shows that the joint commands generated by the framework can be used to drive a physical follower robot in the tested configuration, providing a preliminary example of extending the implementation from real2sim to real2real teleoperation.

## 4 Discussion

This work presents a framework for establishing real2sim bimanual teleoperation systems for the evaluation of simulated robotic tools. Using the proposed framework, an Omni‐to‐Unity bimanual teleoperation system was first established and evaluated, followed by a dVRK‐to‐Unity implementation using a different type of leader robot. Across the reported tasks, the numerical IK solver generated continuous joint trajectories with low Cartesian pose errors, while the measured computation time statistics characterised its performance under the evaluated configurations. Additional experiments illustrated the application of the framework to robotic tool design, an 11‐DOF follower system with haptic feedback, and simulated 7‐DOF redundant robots. A supplementary real2real demonstration was also conducted using a physical 7‐DOF Franka robot. Collectively, these results provide experimental examples of the framework operating with different leader devices, follower kinematics, and teleoperation configurations.

The proposed framework was designed to facilitate integration with different leader robots and simulated follower tools through a common kinematic representation. For a new leader robot, its EE pose is transformed from the leader base frame into the coordinate system defined for the simulated follower, with the corresponding transformation and motion scaling specified within the framework. For a simulated follower, the DH parameters and initial joint configuration are defined in the real2sim kernel and can be modified according to the kinematic structure of the tool. This parameterised formulation allows different tool designs to be incorporated without restructuring the overall framework, as illustrated by the configurations evaluated in Experiments 3–5. In addition, Experiment 6 provides a preliminary example in which the generated joint commands were applied to a physical follower robot, indicating the potential extension of the framework from real2sim to real2real teleoperation when an appropriate robot control interface is available.

For modifications to an existing robotic tool design, such as changes in link lengths or joint types, the corresponding DH parameters can be updated while retaining the remaining framework structure. When the simulated follower is replaced by a kinematically different system, the principal parameters requiring configuration include the DH parameters, leader‐to‐follower transformation matrix, motion scaling factor, and initial joint configuration, as summarised for the 11‐DOF system in Table 5. Joint limits and a corresponding URDF model can additionally be specified when required for joint constraints and visualisation, respectively. In particular, the adjustable initial joint configuration provides a means of defining the correspondence between the initial poses of the leader and follower systems. Because different robotic tools may achieve a given Cartesian pose with different joint configurations, specifying this initial configuration independently allows the pose correspondence to be adapted to the kinematic structure of the follower. Table 6 summarises these characteristics in comparison with selected teleoperation systems reported in the literature.

**TABLE 6:** Comparison of relevant teleoperation systems and their key characteristics.

System	Category	Type	Envir.	Leader	Follower	PMD	IK solver	Haptic FB	Adj.DH
This work	real2sim	Bimanual	Ubuntu; ROS.	6‐DOF Omni; 7‐DOF MTMs.	6‐DOF tool design; 7‐DOF Franka; 11‐DOF robotic system.	√	Numerical	√	√
2011 Ye et al. [24]	real2sim	Manual	MATLAB Simulink	6‐DOF Phantom Premium	6‐DOF robot design	×	Analytical (Robot‐specific)	×	×
2017 Dhivin et al. [26]	real2sim; real2real.	Manual	MATLAB	6‐DOF Omni	5‐DOF Dagu robot	×^b^	Analytical (Robot‐specific)	√	×
2019 Naceri et al. [23]	real2real (With DT)	Manual	Windows; Unreal VR; ROS; Ubuntu; Gazebo.	6‐DOF HMC	6‐DOF UR5	√	Jacobian‐based	×	×
2024 Wang et al. [27]	real2real (With DT)	Manual (With RCM)	ROS	7‐DOF Sigma.7	7‐DOF KUKA iiwa14; 10‐DOF robotic system.	√	Jacobian‐based	√	×
2024 Nasiri et al. [28]	real2real (With DT)	Manual (With RCM)	ROS1; ROS2.	6‐DOF Omni	11‐DOF robotic system	√	Jacobian‐based	×	×
2024 Garcia et al. [29]	real2real (With DT)	bimanual ^a^	Ubuntu	6‐DOF Omni; Joystick.	6‐DOF UR5	×	Weighted QP	√	×

Abbreviations: Adj.DH, adjustable DH parameters; DT, digital twin; Envir., development environment; Haptic FB, haptic feedback; HMC, HTC Vive Pro motion controller; PMD, pose mapping details; QP, quadratic programme; RCM, remote centre of motion.

^a^ here, bimanual teleoperation refers to one hand operating a joystick for 3D orientation control and the other hand operating the Omni stylus for 3D position control.

^b^ only 3D position mapping involved.

The kinematic computations in the proposed framework are implemented independently of a specific visualisation platform. Consequently, the generated joint states can, in principle, be communicated to different simulation or visualisation environments, such as Unity, Blender, MATLAB, V‐REP/CoppeliaSim, or Gazebo, provided that an appropriate communication interface is available. In the implementations presented in this work, ROS was used to communicate the generated joint states to Unity. Alternatively, the robot state can be visualised directly using the ROS‐based visualisation tool RViz when an appropriate URDF model is available. This separation between the real2sim kernel and the visualisation environment reduces the dependence of the kinematic framework on a particular simulation platform.

Mechanical development of robotic tools commonly involves iterative modifications to characteristics such as joint types, number of joints, link dimensions, and internal channels. Some of these modifications alter the kinematic model and therefore require corresponding changes to the DH parameters. By treating the DH table as an adjustable input to the framework, the kinematic model, FK, Jacobian matrix, and numerical IK calculations can be updated according to the specified tool configuration. Experiment 3 illustrates this capability by comparing two tool designs with different link lengths under the same teleoperation input. The same parameterised formulation was also applied to kinematically different serial systems, including the 11‐DOF follower in Experiment 4 and the 7‐DOF Franka robots in Experiment 5. These examples suggest that the formulation can accommodate different serial manipulator configurations when their kinematics can be represented using the DH convention adopted in the framework.

In the dVRK, the orientation mapping between the MTM and PSM is absolute, that is, the orientation of the PSM directly corresponds to that of the MTM. In this work, a relative orientation mapping strategy is adopted to provide greater flexibility in leader‐follower pose correspondence. For example, the user does not need to maintain the EE of the physical leader robot in a strictly vertically downward orientation to obtain a corresponding vertically downward orientation of the simulated robotic tool. When the initial orientations of the leader and follower EEs are identical, the relative orientation mapping used in this work becomes equivalent to an absolute orientation mapping.

Compared with our previous work [30], the numerical IK solver in the present implementation had a moderately higher computational cost. Several implementation differences may contribute to this increase. First, two leader‐follower pairs are processed in the bimanual system. The bimanual implementation also increases the overall system workload, which may contribute to computation time variation depending on resource scheduling. Second, the Jacobian matrix and FK were implemented as externally generated symbolic functions in our previous work, whereas they are computed online from the specified DH parameters in the present framework. Consequently, the required kinematic quantities are reconstructed from the DH representation during numerical IK computation. This introduces additional computation but allows changes in the DH parameters to be incorporated directly without regenerating external symbolic FK or Jacobian functions. As reported in Table 4, the p99 IK computation times remained between 2.4815 and 3.0476 ms across the leader‐follower pairs evaluated in Experiments 1 and 2, providing a quantitative characterisation of the upper‐tail computation time under the reported tasks.

In the present implementation, haptic force commands were transmitted to the Omni devices at 500 Hz. Experiment 4 showed that force‐feedback signals associated with tool‐tissue interactions could be generated and transmitted to the Omni devices during the simulated grasping‐and‐cutting task. However, the fidelity and perceptual quality of the rendered haptic feedback were not quantitatively evaluated in this study. Higher update rates, commonly around 1000 Hz for high‐fidelity haptic rendering, may be desirable for more complex interactions involving, for example, suturing, contact transitions, or collision handling. Haptic rendering is not an intrinsic component of the proposed kinematic framework, and its implementation depends on the capabilities of the selected leader device and simulation environment. Therefore, the framework can be implemented with or without haptic feedback, while the specific force‐rendering method and update rate can be selected according to the application and visualisation platform.

The proposed framework primarily performs kinematic mapping and numerical IK computation and does not itself implement a dynamic robot control architecture. Therefore, a formal control‐stability analysis is outside the scope of the present framework. Nevertheless, the behaviour of the numerical IK solver is important for continuous teleoperation. Across the trajectories evaluated in Experiments 1 and 2, continuous joint solutions were obtained without visually apparent discontinuities in the recorded trajectories, and the corresponding Cartesian pose errors and computation time statistics were quantified in the results. No IK‐related system failures were observed during the reported task executions. These observations provide empirical evidence that the implemented numerical IK solver supported continuous teleoperation for the tested trajectories and system configurations. However, they should not be interpreted as a general proof of solver convergence or stability for arbitrary robotic systems, configurations, or trajectories.

## 5 Limitations and Future Work

The proposed framework focuses on real2sim bimanual teleoperation for evaluating rigid serial robotic tool designs through simulated manipulation tasks. The present work represents an initial step towards a more comprehensive platform for robotic tool design and evaluation. Accordingly, the current implementation has several limitations regarding the types of robotic systems supported, control capabilities, dynamic modelling, and fidelity of surgical task simulation. These limitations also identify directions for future development.

First, the current framework is formulated for rigid serial manipulators and robotic tool designs whose kinematics can be represented using the adopted DH convention. It is therefore not directly applicable to robotic systems with substantially different kinematic representations, such as parallel mechanisms or soft robots. These systems generally require modelling approaches beyond the serial‐link DH formulation used in the present framework. Extending the framework to accommodate alternative kinematic representations and robot structures constitutes an important direction for future work.

Second, the current framework primarily performs leader‐to‐follower pose mapping and numerical IK computation, with the resulting joint configurations provided as commands to the simulated or physical follower. It does not include a dedicated closed‐loop controller for incorporating additional task objectives or constraints. Consequently, capabilities such as singularity avoidance, collision avoidance, manipulability optimization, algorithmic RCM constraints, and hierarchical task prioritisation are not explicitly addressed within the current framework. Future work will integrate a kinematic control layer that can accommodate multiple task objectives and constraints, including optimization‐based approaches such as hierarchical quadratic programming (HQP). Such an extension would enable surgery‐specific constraints to be incorporated into the teleoperation framework, for example, an algorithmically enforced RCM constraint for transoral surgical procedures.

Third, the current framework models the kinematics of the follower systems but does not explicitly incorporate their dynamics. Consequently, dynamic properties such as mass, inertia, friction, actuator characteristics, and external forces are not considered in the current leader‐to‐follower mapping and IK formulation. This limits the framework's ability to represent or evaluate behaviours that depend on robot dynamics and precludes the direct implementation of dynamics‐based control within the present formulation. Future work will investigate the incorporation of dynamic models to support evaluation of dynamic behaviour and the implementation of dynamics‐based control strategies where required.

Fourth, the fidelity of the simulated surgical environment and task interactions remains limited in the present implementation. Because the proposed framework separates the kinematic teleoperation kernel from the visualisation and physics environment, the tissue models and interaction mechanics can be further developed independently of the core framework. Future work will investigate higher‐fidelity tissue models and more complex surgical tasks, including cutting and suturing. The simulated tissue behaviour could also be quantitatively evaluated against experimental biomechanical data using measures such as stress‐strain relationships and tool‐tissue interaction forces. Advanced physics engines and simulation libraries may be incorporated to support these developments. For example, CRESSim‐MPM [38] is a GPU‐accelerated material point method (MPM) library that provides multiple MPM solvers and surgical geometries for simulating procedures including cutting and suturing, and could be integrated as a specialised physics engine within Unity for future surgical simulation studies.

Finally, the experimental evaluation in this study was primarily intended to demonstrate the feasibility and applicability of the proposed framework across several leader‐follower configurations and simulated tasks. The reported experiments were not designed as repeated comparative studies, and statistical analyses of task‐level performance across multiple users or repeated trials were not conducted. Although Cartesian pose errors and IK computation time statistics were quantified for the primary teleoperation experiments, broader evaluation of system performance will require repeated trials, additional task‐level metrics, and extensive user studies. Future work will therefore include systematic evaluations across multiple users, robotic tool configurations, and surgical tasks, with quantitative measures of task performance, system latency, IK failure rates, and teleoperation usability.

## 6 Conclusions

This work presented a compact framework for establishing real2sim bimanual teleoperation systems for the evaluation of robotic tool designs. Using the proposed framework, two bimanual teleoperation systems were implemented: (1) a portable Omni‐to‐Unity system and (2) a dVRK‐to‐Unity system. Both systems were evaluated through simulated bimanual tissue grasping‐and‐cutting tasks. Across the reported experiments, continuous numerical IK solutions were obtained with low Cartesian pose errors, while the IK computation time statistics provided a quantitative characterisation of the computational performance. Additional experiments illustrated the application of the framework to robotic tool design, an 11‐DOF follower system with haptic feedback, and redundant 7‐DOF follower robots. These results provide empirical evidence of the framework's applicability to the leader‐follower configurations and simulated tasks evaluated in this study.

The proposed framework provides a parameterised approach for incorporating different serial robotic tool designs through configurable kinematic parameters, including DH parameters, initial joint configurations, leader‐to‐follower transformations, and motion scaling factors. This formulation can support simulation‐based evaluation and iterative development of robotic tools for applications such as robot‐assisted cleft palate surgery and transoral robotic surgery (TORS). The supplementary real2real experiment further showed that the joint commands generated by the framework could be applied to a physical follower robot in the tested configuration, indicating the potential for extending the framework to real2real teleoperation. Future work will focus on incorporating advanced control strategies and dynamic modelling, improving surgical simulation fidelity, and conducting more systematic experimental and user evaluations.

## Author Contributions

T.Li, S.Z., C.B., T.L. and D.P.: conceptualisation, methodology. T.Li, S.Z., C.B.: data curation, validation. T.Li: formal analysis, visualisation, writing – original draft preparation. T.L. and D.P.: funding acquisition, project administration, supervision. T.Li, S.Z., C.B., T.L. and D.P.: writing – review and editing. All authors have approved the manuscript.

## Funding

This research is supported by the Canadian Institutes of Health Research (CIHR) under Grant 6210101144, and by the Research Training Competition (Restracomp) Fellowship funding from the Research Training Centre (RTC) at The Hospital for Sick Children.

## Ethics Statement

The authors have nothing to report.

## Consent

The authors have nothing to report.

## Conflicts of Interest

The authors declare no conflicts of interest.

## Permission to Reproduce Material From Other Sources

The authors have nothing to report.

## Data Availability

The data that support the findings of this study are available from the corresponding author upon reasonable request.
